# EUGHS NEWS

**DOI:** 10.7189/jogh.06.020204

**Published:** 2016-12

## WHO headquarters: my experiences as an intern – by Ariadne L'Heveder

Photo: Ariadne L’Heveder at the World Health Organisation

“Under the supervision of the team lead of GISRS (Global Influenza Surveillance and Response Systems) and the overall guidance of the head of the Global Influenza Programme, the intern will support activities that directly relate to the finalization of the RSV (respiratory syncytial virus) surveillance strategy.” A somewhat daunting instruction to read following my elation at having secured an internship at the World Health Organisation (WHO) headquarters for the summer before starting my final year of medicine at the University of Edinburgh. However, what started as an overwhelming amount of paperwork, developed into an unforgettable 6 weeks in Geneva.

One of the main reasons I applied to intern at the WHO was because I wanted to gain a sense of how such a vast organization operates on a day–to–day basis, and see whether I could envisage myself working for the WHO given my key interests in global and public health. I was incredibly fortunate to be offered an internship with the Global Influenza Surveillance and Response Systems (GISRS) network, particularly as I joined the team at a very exciting time when they were on the cusp of launching a new pilot for RSV surveillance based on the GISRS platform. RSV is a major cause of global infant mortality and morbidity, especially in low and middle–income countries. Currently however, there is no standardized global surveillance, unlike for influenza virus, where GISRS has successfully carried out surveillance for over 60 years. Thus, the team was in the preliminary stages of establishing what will hopefully become global RSV surveillance, the first step being planning a RSV surveillance pilot. Following a number of meetings to determine the practicalities, epidemiology and laboratory aspects of piloting RSV surveillance, a further meeting: “WHO Technical Meeting on Piloting RSV Surveillance based on the Global Influenza Surveillance and Response System”, which I would help the team prepare for, was arranged to finalise the RSV surveillance strategy before the roll–out of the pilot in September 2016.

I arrived mid–June 2016 and with the meeting starting on 28th June, it was action stations from the get–go! I was assigned a number of tasks to help with the meeting preparations, such as writing the first draft of slides for the WHO speakers’ presentations, and developing a specimen submission form and an accompanying excel data reporting spread–sheet to go into the guidance document for the RSV surveillance pilot. I also became very involved in setting the agenda for the meeting and was amazed by how much my views were respected and helped shape the planning of the meeting; here was a group of people with such a wealth of experience behind them listening to a meagre intern and taking my ideas on board. It must be said that the working environment is very positive at the WHO. While a strict hierarchy inevitably exists, as I’ve seen in hospitals, there is a much more fluid exchange of ideas between colleagues at all levels with a huge respect for others’ opinions which I found very refreshing. Attending the RSV technical meeting was a truly unique opportunity, and I felt so privileged to be surrounded by world experts on RSV, leading public health figures, and the representatives from 14 different countries to be enrolled in the pilot, as well as the WHO staff from headquarters and other regional offices.

Following the meeting there was a great deal to follow up on. One key task was helping to redraft the surveillance document and specimen submission form taking on board the comments made at the meeting. I also began the process of creating a pamphlet for health care professionals who would be involved in the RSV surveillance pilot at the sentinel sites. Sadly 6 weeks was far too short a time period for an internship, with most interns working for at least 2 months, and I had to leave with some work left unfinished. I will however hopefully continue to be involved with GISRS peripherally from Edinburgh.

A particularly sad part about leaving was having to say bye to all the incredible people I met during my internship. As well as the staff, I met some wonderful people interning at the WHO and other UN organisations in Geneva. Being surrounding by a community of like–minded people with the most inspiring ambitions for their futures was truly electric. From weekend trips around Europe, hiking the Alps, and BBQs by Lake Geneva, to attending meetings and fascinating seminars together at the WHO, I made some unforgettable memories and had my mind–opened to lots of new public and global health concepts.

It was an utterly unique experience to be part of the team working on the preliminary steps that will hopefully lead to global RSV surveillance. This internship has most certainly bolstered my enthusiasm for global health and has provided me with new skills and key knowledge in virus surveillance and pandemic response, as well as some great contacts and lovely memories with new friends. I cannot thank Professor Harry Campbell and Dr Wenqing Zhang’s team at WHO enough for providing me with this opportunity.

Photo: The 2016 interns (including Ariadne L’Heveder) with Dr Margaret Chan, the WHO Director–General

